# Liver resection in patients with a history of local ablation for hepatocellular carcinoma has the risk of poor survival and serosal invasion

**DOI:** 10.1002/ags3.12872

**Published:** 2024-11-18

**Authors:** Yusuke Nishi, Katsunori Sakamoto, Mio Uraoka, Tomoyuki Nagaoka, Masahiko Honjo, Kei Tamura, Naotake Funamizu, Kohei Ogawa, Yasutsugu Takada, Yuzo Umeda

**Affiliations:** ^1^ Department of Hepato‐Biliary‐Pancreatic and Breast Surgery Ehime University Graduate School of Medicine Toon Ehime Japan

**Keywords:** hepatectomy, hepatocellular carcinoma, radiofrequency ablation, recurrence

## Abstract

**Aim:**

The aim was to evaluate the impact of previous local ablation (LA) on long‐term prognoses and tumor histopathological findings following hepatectomy for hepatocellular carcinoma (HCC).

**Methods:**

This retrospective study used data from patients who underwent initial hepatectomy for HCC at Ehime University Hospital between October 2003 and July 2021. Using data from a total of 234 patients, after excluding patients with distant metastasis or macroscopic residual tumors and patients with mixed HCC, a group of 39 patients who underwent post‐ablation liver resection (PALR) was compared with a group of 195 non‐PALR patients with respect to patient characteristics, perioperative data, pathological findings, and outcomes.

**Results:**

Number of tumors was significantly greater and diameter of tumor was smaller in PALR group than those of non‐PALR group. Both overall survival (OS) and recurrence‐free survival (RFS) were significantly poor in PALR group than those of non‐PALR (5‐year OS 54.1% vs. 66.9%, *p* = 0.024; 5‐year RFS 24.7% vs. 37.0%, *p* = 0.019). However, PALR was not selected as independent prognosticator in multivariate analyses. In PALR group, tumor size ≥3 cm was sole independent prognosticator in multivariate analyses. Multivariate analysis showed that PALR [odds ratio (OR), 8.989; 95% confidence interval (CI), 2.807–28.787], alpha‐fetoprotein level >40 ng/mL (OR, 2.923; 95% CI, 1.063–8.035), and des‐γ‐carboxyprothrombin level >170 mAU/mL (OR, 5.164; 95% CI, 1.622–16.438) were independent predictors of pathological serosal invasion.

**Conclusions:**

Hepatectomy for patients with history of LA for HCC had a risk of serosal invasion and poor survival.

## Graphical abstract

Local ablation therapy is one of the feasible treatment options for patients with hepatocellular carcinoma. However, recurrence after local ablation therapy is often seen in clinical practice. We found that hepatectomy for the recurrence after local ablation therapy has the risk of poor prognosis due to serosal invasion of the liver.

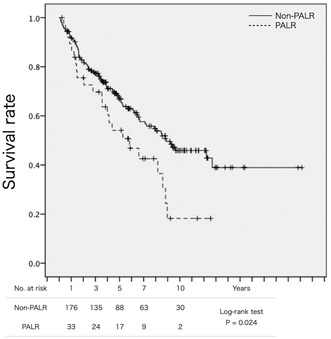

## 1 INTRODUCTION

Liver cancer is the fourth most common cause of cancer‐related death in the world, and hepatocellular carcinoma (HCC) accounts for most primary liver cancers.^1^ For the radical treatment of HCCs, local treatments including surgical resection and local ablation (LA) therapy are considered essential to achieve good outcomes.^2^ Surgical resection and LA showed comparable outcomes for selected patients with HCC.^3^ LA is a good treatment option for patients with very early or early stage disease [Barcelona Clinic Liver Cancer (BCLC)‐0 or BCLA‐A, respectively] according to the 2022 BCLC staging and treatment strategy.^4^ A study that compared hepatectomy and radiofrequency ablation (RFA) in HCC with a Child–Pugh score ≤7 points, tumor diameter ≤3 cm, and ≤3 tumors found no difference between the two treatments with respect to long‐term outcomes,^4^ and there have been several other similar reports.^5^, ^6^, ^7^ The Japanese clinical practice guidelines for HCC also recommend hepatectomy and LA therapy as equally effective in patients with Child–Pugh classification A or B HCC without extrahepatic metastasis or vascular invasion, ≤3 tumors, and tumor diameter ≤3 cm.^8^ However, the HCC recurrence rate is high even after these radical treatments, with the recurrence rate reaching 70% 5 years after hepatectomy and the cumulative recurrence rate reaching 72.0% 5 years after LA.^9^, ^10^ Consequently, resection and LA are also frequently performed for recurrent HCC after radical treatment because the Japanese guidelines indicate that treatment for recurrent HCC is the same as that for initial HCC.^8^ No significant difference was reportedly found between the long‐term outcomes of hepatectomy and RFA for HCC that recurred after local treatment.^11^, ^12^, ^13^, ^14^ Another study found that the outcome of hepatectomy performed for local recurrence after LA was similar to the outcome of initial hepatectomy.^15^ In contrast, recently, Park et al. reported that salvage hepatectomy for local recurrences after LA showed poorer long‐term prognosis compared with hepatectomy for incipient HCC.^16^ However, few studies have compared the characteristics and post‐hepatectomy outcomes between local recurrent lesions occurring after LA and incipient HCC.

The objective of this study was to compare how the presence of a history of LA affects tumor histopathology and prognosis after hepatectomy in patients who underwent initial hepatectomy for HCC.

## 2 METHODS

### 2.1 Patients and data collection

**FIGURE 1:**
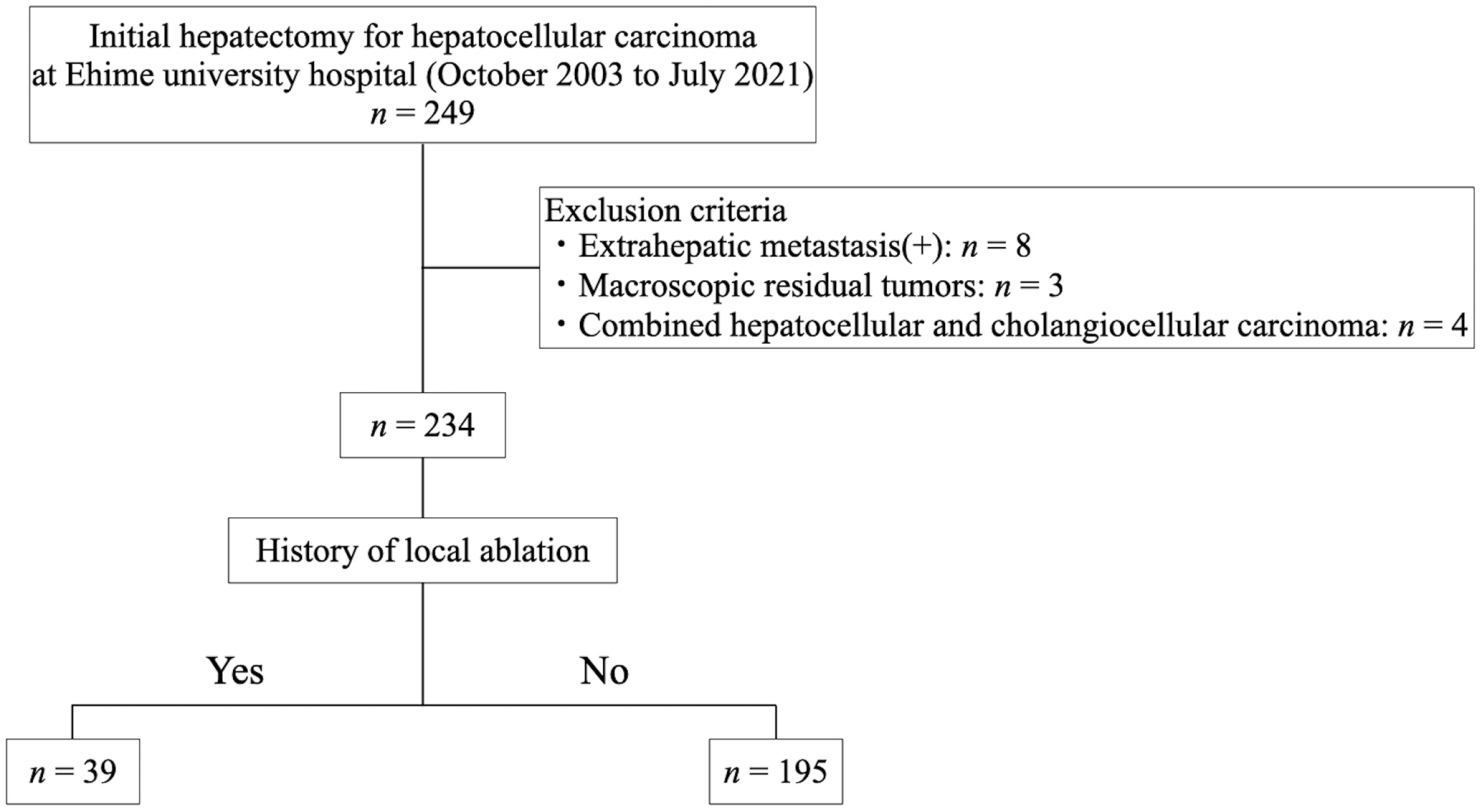
Patient flow chart.

This retrospective study used data from 249 patients who underwent initial hepatectomy for HCC at Ehime University Hospital (Toon, Japan) between October 2003 and July 2021. Excluded were 11 patients with distant metastasis or macroscopic residual tumors (R2 resection) and four patients with combined HCC and cholangiocarcinoma. The remaining 234 patients were ultimately included in the study. Patients who, during the resection procedure, underwent curative LA for lesions were not excluded. Patients who had history of LA were defined as the post‐ablation liver resection (PALR) group (Figure 1). LA in the present study included either percutaneous ethanol injection therapy (PEIT) or RFA; no patients underwent microwave ablation in the present cohort. The non‐PALR group was defined as the remaining patients (Figure 1).

The median follow‐up period was 55.0 months (range, 1.1–241.4 months). With the 234 patients classified as PALR‐group or non‐PALR‐group patients, the patients' characteristics, preoperative examinations, postoperative outcomes, and pathological findings of the two groups were evaluated. In addition, the factors that affected overall survival (OS), and recurrence‐free survival (RFS) were examined by univariate and multivariate analyses. Prognostic factors specific to the PALR group were evaluated as well.

### 2.2 Patient follow‐up examinations and treatment strategies for recurrence

Follow‐up was performed at our hospital once every 1–3 months after hepatectomy. Blood biochemistry tests, including alpha‐fetoprotein (AFP) and des‐γ‐carboxyprothrombin (DCP) levels, were performed at each outpatient examination. Radiological imaging was performed every 3–6 months. Treatments for recurrence included repeat hepatectomy, LA, transcatheter arterial chemo‐embolization (TACE), or systemic chemotherapy including molecularly targeted drug therapy, as needed.

### 2.3 Statistical analysis

Continuous variables, presented as median and range values, were compared using the Mann–Whitney *U*‐test. Categorical variables, presented as numbers and percentages, were compared by the *χ* ^2^‐test or Fisher's exact test. Survival curves were generated by the Kaplan–Meier method and compared by the log‐rank test. RFS was defined as the period from completion of hepatectomy to the detection of recurrence or death of the patient. Only significant variables from univariate analyses were adopted for multivariate analyses. Cut‐off values of the continuous variables were determined using receiver‐operating characteristic (ROC) curves. Multivariate analyses were carried out using Cox proportional hazards modeling or logistic regression analysis. All tests were two‐sided, and values of *p* < 0.05 were considered to indicate significance. All statistical analyses were performed with EZR (Saitama Medical Center, Jichi Medical University, Saitama, Japan), which is a graphical user interface for R (The R Foundation for Statistical Computing, Vienna, Austria, version 4.2.3). More precisely, it is a modified version of R commander (version 2.8‐0) designed to add statistical functions frequently used in biostatistics.^17^

### 2.4 Ethical considerations

This study was approved by the institutional review board at Ehime University Hospital (approval no. 2211013) and was conducted in accordance with the ethical standards laid down in the 1995 version of the Declaration of Helsinki (as revised in Brazil 2013). Informed consent was by the opt‐out principle, with general information posted on the study website, along with the opportunity to refuse participation.

## 3 RESULTS

Of the 234 study patients, 39 (16.7%) were in the PALR group. LA was PEIT in 10 patients (25.6%) and RFA in 29 patients (74.4%). The median time from the last previous LA to hepatectomy was 405 days (21 to 6661 days), and the median number of LA therapies before hepatectomy was three.^1^, ^2^, ^3^, ^4^, ^5^, ^6^, ^7^, ^8^, ^9^, ^10^ There was no within 90‐day in‐hospital mortality after hepatectomy in the present cohort.

The differences in patients' characteristics, preoperative examinations, and postoperative outcomes between the PALR and non‐PALR groups are shown in Table 1. Although the albumin‐bilirubin (ALBI) grade was significantly higher in the PALR group than that of non‐PALR group (*p* = 0.016), Child–Pugh classification showed no significant difference between both groups (*p* = 0.276). Although viral hepatitis was significantly more common in the PALR group (*p* < 0.001), the rate of sustained virological response (SVR) in hepatitis C patients showed no significant difference (*p* = 0.573). Preoperative AFP levels showed no significant difference between both groups (*p* = 0.273) but the DCP levels were significantly lower in the PALR group than that of non‐PALR groups (*p* = 0.028). Surgical duration was significantly longer in the PALR group than non‐PALR group (*p* = 0.037) but frequency of anatomical liver resection was significantly greater in the PALR group than non‐PALR group (*p* = 0.004). Examination of histopathological factors showed that tumor size was significantly larger in the non‐PALR group (*p* < 0.001). Number of tumors was significantly greater in the PALR group (*p* = 0.021). There were no patients with pathological positive surgical margins due to vascular invasion or serosal invasion in both groups. Patients with vascular invasion, serosal invasion, and fibrosis progression in non‐malignant areas were significantly more common in the PALR group. However, no significant differences were seen in the macroscopic type and degree of histological differentiation.

**TABLE 1:** Patients' characteristics, perioperative data, and pathological findings.

	PALR	Non‐PALR	*p*‐value
	*n* = 39	*n* = 195	
Clinical findings			
Age, years	70 (56–84)	70 (31–89)	0.456
Sex, male	24 (61.5%)	149 (76.4%)	0.053
ALBI score	−2.26 (−3.67–‐1.67)	−2.51 (−3.30–‐0.99)	0.016
ALBI grade			
1	13 (33.3%)	80 (41.0%)	0.290
2	26 (66.7%)	111 (56.9%)	
3	0 (0.0%)	4 (2.1%)	
Child–Pugh classification			
A	38 (97.4%)	177 (90.8%)	0.276
B	1 (2.6%)	17 (8.7%)	
C	0 (0.0%)	1 (0.5%)	
Etiology			
HBV	9 (23.1%)	40 (20.5%)	0.001
HCV	28 (71.8%)	76 (39.0%)	
Others	2 (5.1%)	79 (40.5%)	
Viral hepatitis (HBV or HCV)	37 (94.9%)	116 (59.5%)	<0.001
SVR rate^a^	11/28 (39.3%)	25/75 (33.3%)	0.573
Preoperative TACE treatment	2 (5.4%)	16 (8.2%)	0.428
Time from initial ablation to resection, day	1720 (84–6661)	–	–
Time from last ablation to resection, day	405 (21–6661)	–	–
Number of ablations	3 (1–10)	–	–
Type of ablation, RFA/PEIT	29 (74.4%)/10 (25.6%)	–	–
Hepatectomy for same segment recurrence after ablation	27 (69.2%)	–	–
AFP, ng/mL	11.0 (2.0–3924.0)	7.8 (0.9–296660.0)	0.273
DCP, mAU/mL	46.0 (14.0–24235.0)	114.0 (11.0–538236.0)	0.028
Number of tumors	1 (1–5)	1 (1–5)	0.031
Maximum diameter of the tumors, cm	2.3 (0.6–6.0)	3.8 (0.7–20.0)	<0.001
Perioperative findings			
Operating time, min	475 (158–556)	374 (100–650)	0.037
Blood loss volume, mL	545 (0–3824)	458 (0–10 000)	0.639
Anatomical liver resection	19 (48.7%)	141 (72.3%)	0.004
Red cell transfusion	15 (39.5%)	58 (29.7%)	0.237
Postoperative complications (≥C–D III)	8 (20.5%)	37 (19.0%)	0.562
Postoperative hospital stay, days	16 (4–81)	16 (4–168)	0.824
Pathological findings			
Number of tumors	1 (1–5)	1 (1–5)	0.021
Tumor size, cm	2.4 (0.5–11.0)	3.5 (0.4–18.0)	< 0.001
Multiple lesions	15 (38.5%)	42 (21.5%)	0.025
Surgical margin width, mm	2.5 (0.0–21.0)	7.0 (0.0–50.0)	0.100
Surgical margin 0 mm	1 (2.6%)	3 (1.5%)	0.520
Macroscopic type^b^			
Simple nodular type	22 (56.4%)	111 (56.9%)	0.174
Simple nodular with extranodular growth	3 (7.7%)	28 (14.4%)	
Confluent multinodular type	11 (28.2%)	52 (26.7%)	
Others	3 (7.7%)	4 (2.0%)	
Differentiation^b^			
Well/moderate/poor/unknown	5 (12.8%)/26 (66.7%)/5 (12.8%)/3 (7.7%)	24 (12.3%)/140 (71.8%)/14 (7.2%)/17 (8.7%)	0.804
Invasion of hepatic artery^b^	0 (0.0%)	0 (0.0%)	–
Invasion of portal vein^b^	18 (46.2%)	54 (27.7%)	0.023
vp0/vp1/vp2/vp3/vp4	19 (50.0%)/13 (34.2%)/1 (2.6%)/5 (13.2%)/0	140 (71.8%)/47 (24.1%)/2 (1.0%)/4 (2.1%)/2 (1.0%)	0.017
Invasion of hepatic vein^b^	2 (5.1%)	15 (8.1%)	0.437
vv0/vv1/vv2/vv3	36 (94.7%)/0/3 (5.3%)/0	181 (92.8%)/11 (5.6%) /1 (0.5%)/2 (1.0%)	0.038
Invasion of bile duct^b^	3 (7.7%)	3 (1.5%)	0.060
b0/b1/b2/b3/b4	35 (92.1%)/2 (5.3%)/0/1 (2.6%)/0	192 (98.5%)/1 (0.5%)/2 (1.0%)/0/0	0.041
Serosal invasion^b^	10 (25.6%)	12 (6.2%)	0.001
s0/s1/s2/s3	29 (74.4%)/5 (12.8%)/4 (10.3%)/1 (2.6%)	183 (93.8%)/6 (3.1%)/3 (1.5%)/3 (1.5%)	0.006
Degree of liver fibrosis^b^			
f0/f1/f2/f3/f4	2 (5.6%)/1 (2.8%)/2 (5.6%)/3 (8.3%)/28 (77.8%)	28 (14.7%)/44 (23.0%)/26 (13.6%)/24 (12.6%)/69 (36.1%)	<0.001

*Note*: Continuous variables are presented as median (range) values. Categorical variables are presented as *n* (%) values. Well, well‐differentiated tubular adenocarcinoma; mod, moderately differentiated tubular adenocarcinoma; poor, poorly differentiated tubular adenocarcinoma; vp0, invasion of (or tumor thrombus in) portal vein undetected; vp1, invasion of (or tumor thrombus in) distal to second order branches (second order branches not included) of the portal vein detected; vp2, invasion of (or tumor thrombus in) second order branches of the portal vein detected; vp3, invasion of (or tumor thrombus in) first order branches of the portal vein detected; vp4, invasion of (or tumor thrombus in) the main trunk of the portal vein and/or contralateral portal vein branch to the primarily involved lobe detected; vv0, invasion of (or tumor thrombus in) the hepatic vein undetected; vv1, invasion of (or tumor thrombus in) peripheral branches of the hepatic vein detected; vv2, invasion of (or tumor thrombus in) the right, middle, or left hepatic vein, the inferior right hepatic vein, or the short hepatic vein detected; vv3, invasion of (or tumor thrombus in) the inferior vena cava detected; b0, invasion of the bile duct undetected; b1, invasion of (or tumor thrombus in) third order or more peripheral branches of the bile duct, but not of second order branches, detected; b2, invasion of (or tumor thrombus in) second order branches of the bile duct detected; b3, invasion of (or tumor thrombus in) first order branches of the bile duct detected; b4, invasion of (or tumor thrombus in) the common hepatic duct detected; s0, tumor invasion of serosa undetected; s1, tumor invasion of serosa detected; s2, tumor invasion of adjacent organs detected; s3, tumor rupture with intraperitoneal bleeding detected; f0, no fibrosis; f1, fibrous expansion of portal tract; f2, fibrous septa formation, usually incomplete; f3, bridging fibrosis formation accompanying lobular distortion; f4, cirrhosis.

Abbreviations: AFP, α‐fetoprotein; ALBI, albumin‐bilirubin; C–D, Clavien–Dindo classification; DCP, des‐γ‐carboxyprothrombin; HBV, hepatitis B virus; HCV, hepatitis C virus; PALR, post‐ablation liver resection; PEIT, percutaneous ethanol injection therapy; RFA, radiofrequency ablation; SVR, sustained virological response; TACE, transcatheter arterial chemo‐embolization.

^a^ Of patients with positive HCV.

^b^ Defined by the General Rules for Clinical and Pathological Study of Primary Liver Cancer in Japan (6th edition).

**FIGURE 2:**
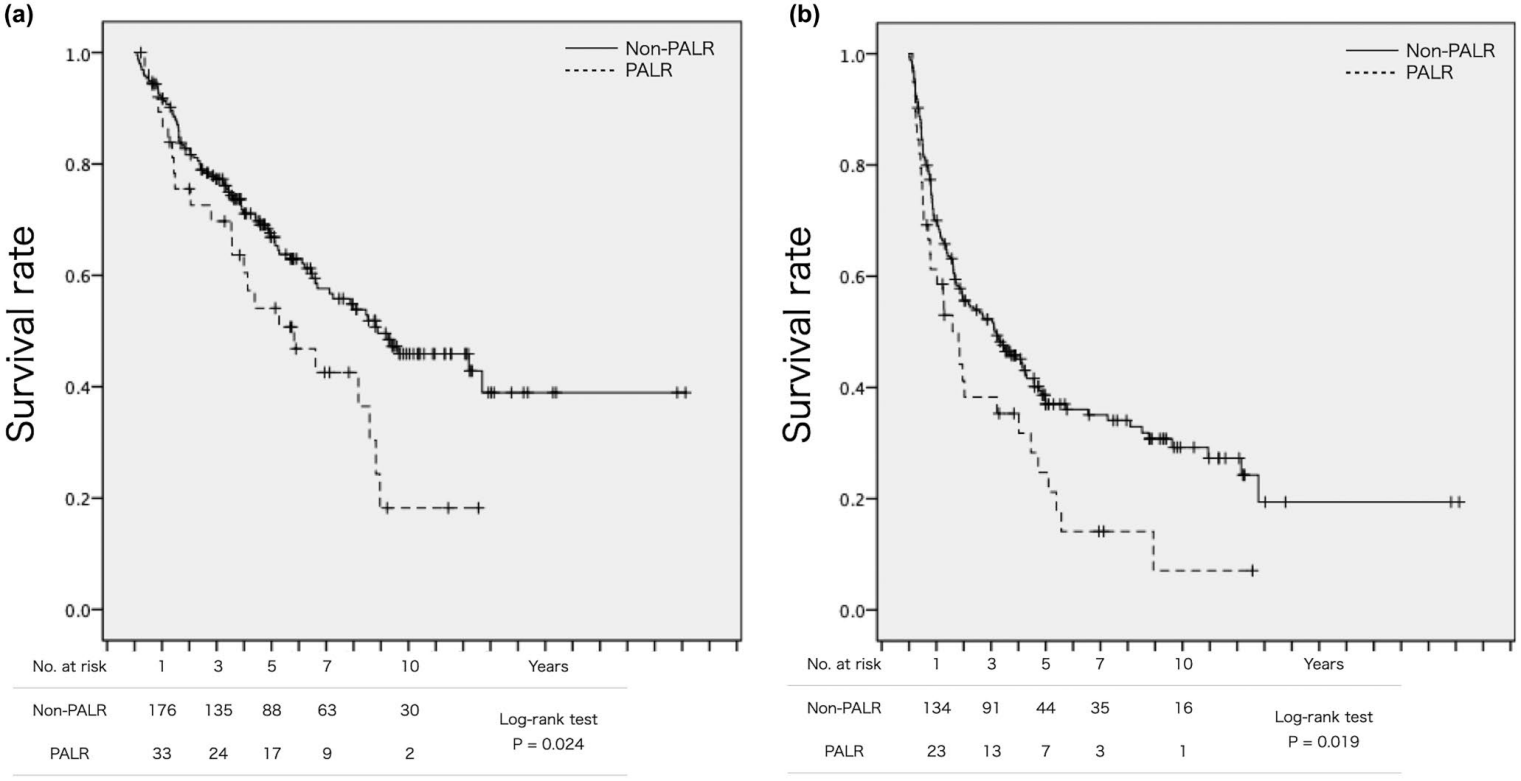
Survival after liver resection for hepatocellular carcinoma in the non‐PALR and PALR groups. (A) Overall survival. Five‐year overall survival rate in PALR group was 66.9% and 54.1% in non‐PALR group (*p* = 0.024). (B) recurrence‐free survival. Five‐year recurrence‐free survival rate in PALR group was 37.0% and 24.7% in non‐PALR group (*p* = 0.019). PALR, post‐ablation liver resection.

PALR groups showed significantly poor survival after hepatectomy compared with non‐PALR group (Figure 2). Multivariate analysis showed high DCP level, vascular invasion, and serosal invasion to be independent risk factors related to OS after hepatectomy (Table 2). With regard to RFS, multivariate analysis showed high ALBI grade, tumor size, the presence of multiple tumors, serosal invasion, and background liver fibrosis to be independent risk factors (Table 3).

**TABLE 2:** Univariate and multivariate analyses for overall survival after hepatectomy.

	Univariate analysis			Multivariate analysis		
	*n*	MST, *m*	*p*‐value	Hazard ratio	95% CI	*p*‐value
Age, year						
≥70	123	98.1	0.859	–	–	–
<70	111	103.1				
Sex						
Male	173	95.9	0.474	–	–	–
Female	61	106.6				
ALBI grade						
≥2	141	79.4	0.014	1.491	0.952–2.335	0.081
1	93	NR				
AFP, ng/mL						
>15	96	63.3	0.003	1.350	0.879–2.072	0.170
≤15	138	103.1				
DCP, mAU/mL						
>70	123	73.4	0.003	1.525	1.007–2.309	0.046
≤70	111	152.3				
PALR						
Yes	39	69.9	0.024	1.102	0.648–1.875	0.720
No	195	106.6				
Anatomical liver resection						
No	74	101.5	0.691	–	–	–
Yes	160	102.2				
Red cell transfusion						
Yes	73	79.2	0.179	–	–	–
No	161	112.9				
Postoperative complication^a^						
Yes	45	46.8	0.012	1.357	0.850–2.165	0.201
No	189	105.8				
Tumor size						
>3	116	80.2	0.071	–	–	–
≤3	118	107.6				
Number of tumors						
Multiple	57	61.4	0.051	–	–	–
Solitary	177	106.6				
Histological differentiation^b^						
Poor	19	61.6	0.341	–	–	–
Others	215	102.2				
Vascular invasion^b^						
Yes	76	42.4	<0.001	1.845	1.187–2.869	0.007
No	158	111.1				
Serosal invasion^b^						
Yes	22	39.4	<0.001	2.458	1.298–4.656	0.006
No	212	106.6				
Degree of liver fibrosis^b^						
f3/f4	124	77.4	0.012	1.542	0.996–2.387	0.052
f0–f2	104	152.3				

Abbreviations: AFP, α‐fetoprotein; CI, confidence interval; DCP, des‐γ‐carboxyprothrombin; f0, no fibrosis; f1, fibrous expansion of portal tract; f2, fibrous septa formation, usually incomplete; f3, bridging fibrosis formation accompanying lobular distortion; f4, cirrhosis; MST, median survival time; NR, not reached; PALR, post‐ablation liver resection.

^a^ Defined as Clavien–Dindo classification ≥III.

^b^ Defined by the General Rules for Clinical and Pathological Study of Primary Liver Cancer in Japan (6th edition).

**TABLE 3:** Univariate and multivariate analyses for recurrence‐free survival after hepatectomy.

	Univariate analysis			Multivariate analysis		
	*n*	MST, m	*p*‐value	Hazard ratio	95% CI	*p*‐value
Age, year						
≥70	123	30.9	0.906	–	–	–
<70	111	37.3				
Sex						
Male	173	23.4	0.108	–	–	–
Female	61	51.2				
ALBI grade						
≥2	141	21.7	<0.001	1.520	1.050–2.201	0.027
1	93	57.3				
AFP, ng/mL						
>15	96	23.4	0.089	–	–	–
≤15	138	41.9				
DCP, mAU/mL						
>70	123	19.3	0.002	1.217	0.841–1.759	0.298
≤70	111	51.2				
PALR						
Yes	39	21.7	0.019	0.179	0.730–1.905	0.501
No	195	37.5				
Anatomical liver resection						
No	74	26.0	0.520	–	–	–
Yes	160	36.5				
Red cell transfusion						
Yes	73	16.7	0.011	1.008	0.700–1.451	0.967
No	161	48.2				
Postoperative complication^a^						
Yes	45	13.9	0.001	1.152	0.763–1.741	0.500
No	189	44.5				
Tumor size, cm						
≥3	116	19.9	0.047	2.058	1.388–3.050	<0.001
<3	118	47.0				
Number of tumors						
Multiple	57	13.8	<0.001	1.581	1.071–2.336	0.021
Solitary	177	48.2				
Differentiation^b^						
Poor	19	13.9	0.047	1.411	0.769–2.590	0.267
Others	215	38.5				
Vascular invasion^b^						
Yes	76	10.2	<0.001	1.390	0.941–2.053	0.098
No	158	48.2				
Serosal invasion^b^						
Yes	22	9.1	<0.001	2.530	1.406–4.552	0.002
No	212	39.5				
Degree of liver fibrosis^b^						
f3/f4	124	19.6	<0.001	2.414	1.635–3.564	<0.001
f0–f2	104	97.2				

Abbreviations: AFP, α‐fetoprotein; CI, confidence interval; DCP, des‐γ‐carboxyprothrombin; f0, no fibrosis; f1, fibrous expansion of portal tract; f2, fibrous septa formation, usually incomplete; f3, bridging fibrosis formation accompanying lobular distortion; f4, cirrhosis; MST, median survival time; PALR, post‐ablation liver resection; poor, poorly differentiated tubular adenocarcinoma.

^a^ Defined as Clavien–Dindo classification ≥III.

^b^ Defined by the General Rules for Clinical and Pathological Study of Primary Liver Cancer in Japan (6th edition).

Table 4 shows prognostic factors specific to the PALR group. The ALBI grade, anatomical resection, red cell transfusion, postoperative complication, number of tumors, histological differentiation, vascular invasion, and degree of liver fibrosis showed no significant impact both for OS and RFS. In addition, features of ablation therapy such as time from last/initial ablation, number of ablations, and hepatectomy for same segment recurrence showed no significant impact for prognosis. However, RFA showed poor OS than PEIT in univariate analysis (Table 4). Multivariate analysis showed that tumor size more than 3 cm was an independent predictor both for OS and RFS (Table 4).

**TABLE 4:** Univariate and multivariate analyses for prognoses after hepatectomy in the patients with history of local ablation (*n* = 39).

	Univariate analysis			Multivariate analysis		
	*n*	MST, m	*p*‐value	Hazard ratio	95% CI	*p*‐value
Overall survival						
DCP, mAU/mL						
>400	8	14.7	0.030	2.252	0.636–8.000	0.208
≤400	31	98.1				
Type of ablation						
RFA	29	49.5	0.028	2.849	0.937–8.621	0.065
PEIT	10	105.8				
Time from last LA						
<1 year	21	69.9	0.530	—	—	—
≥1 year	18	98.1				
Hepatectomy for same segment recurrence after LA						
No	12	42.7	0.235	—	—	—
Yes	27	98.1				
Number of LA before hepatectomy						
≥2	26	52.6	0.419	—	—	—
1	13	79.2				
Tumor size, cm						
≥3	10	12.3	<0.001	6.327	2.484–16.115	<0.001
<3	29	103.1				
Serosal invasion^a^						
Yes	10	42.4	0.023	1.239	0.367–4.180	0.730
No	29	98.1				
Recurrence‐free survival						
DCP, mAU/mL						
>40	21	9.3	0.032	2.113	1.006–4.439	0.048
≤40	18	53.6				
Type of ablation						
RFA	29	15.0	0.210	—	—	—
PEIT	10	53.6				
Time from last LA						
<1 year	21	19.1	0.836	—	—	—
≥1 year	18	22.2				
Hepatectomy for same segment recurrence after LA						
No	12	15.0	0.412	—	—	—
Yes	27	21.7				
Number of LA before hepatectomy						
≥2	26	19.1	0.093	—	—	—
1	13	38.6				
Tumor size, cm						
≥3	10	5.3	0.021	2.810	1.053–7.498	0.039
<3	29	23.5				
Serosal invasion^a^						
Yes	10	2.7	0.012	1.881	0.713–4.961	0.202
No	29	15.7				

Abbreviations: CI, confidence interval; DCP, des‐γ‐carboxyprothrombin; MST, median survival time; NR, not reached; PEIT, percutaneous ethanol injection therapy; RFA, radiofrequency ablation.

^a^ Defined by the General Rules for Clinical and Pathological Study of Primary Liver Cancer in Japan (6th edition).

Multivariate analysis showed that AFP level >40 mAU/mL (odds ratio [OR], 2.923; 95% confidence interval [CI], 1.063–8.035; *p* = 0.038), DCP level >170 mAU/mL (OR, 5.164; 95% CI, 1.622–16.438; *p* = 0.005), and PALR (OR, 8.989; 95% CI, 2.807–28.787; *p* < 0.001) were independent risk factors for pathological serosal invasion (Table 5).

**TABLE 5:** Univariate and multivariate analyses of the risk factors for pathological serosal invasion.

		Univariate analysis			Multivariate analysis		
		Serosal invasion (+)	Serosal invasion (−)	*p*‐value	Odds ratio	95% CI	*p*‐value
		*n* = 22	*n* = 212				
AFP, ng/mL	>40	12 (54.5%)	55 (25.9%)	0.005	2.923	1.063–8.035	0.038
DCP, mAU/mL	>170	15 (68.2%)	79 (37.3%)	0.005	5.164	1.622–16.438	0.005
History of LA	Yes	10 (45.5%)	29 (13.7%)	0.001	8.989	2.807–28.787	< 0.001
Tumor size, cm	≥3	10 (45.5%)	106 (50.0%)	0.685			
Number of tumors	Multiple	9 (40.9%)	48 (22.6%)	0.057			
Differentiation	Poor	3 (13.6%)	16 (7.5%)	0.259			
Vascular invasion^a^	Positive	13 (59.1%)	63 (29.7%)	0.005	1.260	0.439–3.615	0.668
Degree of liver fibrosis^a^	f3/f4	11 (61.1%)	113 (53.8%)	0.551			

*Note*: Categorical variables are presented as *n* (%) values.

^a^ Defined by the General Rules for Clinical and Pathological Study of Primary Liver Cancer in Japan (6th edition).

Abbreviations: AFP, α‐fetoprotein; CI, confidence interval; DCP, des‐γ‐carboxyprothrombin; f3, bridging fibrosis formation accompanying lobular distortion; f4, cirrhosis; LA, local ablation; poor, poorly differentiated tubular adenocarcinoma.

Table 6 shows the detailed data for recurrence after hepatic resection. Although the recurrence was more frequently seen in the PALR group (76.9%) compared with the non‐PALR group (54.9%, *p* = 0.011), type of recurrences showed no significant difference between both groups. More aggressive intrahepatic recurrence (more than three lesions) was significantly more common in the serosal invasion group than that of no serosal invasion group (41.2% vs. 17.1%, *p* = 0.034).

**TABLE 6:** Features of recurrence after hepatectomy.

Recurrence	PALR	Non‐PALR	*p*‐value
	30 (76.9%)	107 (54.9%)	0.011
Type of recurrence			
Intrahepatic	28/30 (93.3%)	95/107 (88.8%)	0.368
Intrahepatic (alone)	23/30 (76.7%)	77/107 (72.0%)	0.608
Intrahepatic, more than 3 lesion	4/30 (13.3%)	24/107 (22.9%)	0.257
Lung	2/30 (6.7%)	12/107 (11.2%)	0.368
Lymph nodes	1/30 (3.3%)	10/107 (9.3%)	0.258
Peritoneal dissemination	0/30 (0.0%)	5/107 (4.7%)	0.285
Others^a^	0/30 (0.0%)	7/107 (6.5%)	0.170
Multiple organ	2/30 (6.7%)	15/107 (16.5%)	0.149

	Serosal invasion (+)	Serosal invasion (−)	*p*‐value
Recurrence	17 (77.3%)	120 (56.6%)	0.061
Type of recurrence			
Intrahepatic	15/17 (88.2%)	108/120 (90.0%)	0.544
Intrahepatic (alone)	9/17 (52.9%)	91/120 (75.8%)	0.049
Intrahepatic, more than 3 lesion	7/17 (41.2%)	21/120 (17.1%)	0.034
Lung	5/17 (29.4%)	9/120 (7.5%)	0.016
Lymph nodes	0/17 (0.0%)	11/120 (9.2%)	0.329
Peritoneal dissemination	1/17 (5.9%)	4/120 (3.3%)	0.490
Others^a^	3/17 (17.6%)	4/120 (3.3%)	0.041
Multiple organ	4/17 (26.7%)	13/120 (12.3%)	0.136

*Note*: Categorical variables are presented as *n* (%) values.

Abbreviation: PALR, post‐ablation liver resection.

^a^ One case of right adrenal metastasis and six cases of bone metastases. The case of right adrenal metastasis was the patient with positive serosal invasion.

## 4 DISCUSSION

In clinical practice, patients with HCC who have good hepatic reserve sometimes have already undergone LA when hepatectomy is considered, and much remains uncertain regarding the oncological impact of these previous treatments. Orimo et al. divided patients according to whether they had previously undergone treatments such as RFA and/or TACE and compared the pathology results at the time of hepatectomy. They found that the presence of multiple tumors, poor differentiation, and portal vein invasion were significantly more common in the previously treated group.^13^ However, their report included a low LA ratio (32%) compared with the present study (100%).^13^ Arii et al. reported that, in patients who had undergone repeat hepatectomy for recurrent HCC after hepatectomy, cancer infiltration into the tumor capsule was significantly more common in patients with intrahepatic metastases than in patients with multicentric carcinogenesis.^18^ However, there have been limited reports of the effects on the characteristics of recurrent lesions and post‐hepatectomy outcomes when the only previous treatment was limited to LA.

Sugo et al. reported that the salvage hepatectomy group for local recurrence after LA showed equivalent short‐ and long‐term outcomes compared with the initial hepatectomy group.^15^ However, although OS showed no significant difference between both groups, the results of disease‐free survival after hepatectomy showed a worse result in previous LA group.^15^ In the present study, although the history of LA was not selected as an independent predictor of long‐term prognosis, both the OS and RFS showed worse survival in the PALR group than those of non‐PALR group. Recently, Park et al. reported that salvage hepatectomy for local recurrences after LA showed similar short‐term outcome compared with initial hepatectomy but the long‐term prognosis was poorer as same as the present study.^16^ The report suggested that local recurrences after LA therapy showed more aggressive behavior and extensive resection are necessary to prevent re‐recurrence.^16^ The poor prognosis in the PALR group may be due to the oncological consequences of the previous LA treatments. It has been surmised that the puncture needle used for LA may seed tumors in the puncture track; that ablation increases intra‐tumoral pressure, resulting in tumor cell dislodgement; and that factors such as insufficient heat stress change the nature of the HCC.^19^, ^20^, ^21^, ^22^, ^23^ These factors may have played some role in worse prognosis after hepatectomy. It has been reported that incomplete RFA showed poor outcomes in HCC.^24^

In contrast to the reports from Sugo et al. and Park et al., patients background was different because the present study included not only the patients with local recurrence after LA but also the patients with different segment recurrences. When the patients were limited to same segment recurrence after LA, though not significant, the long‐term outcome was worse in PALR group (5‐year OS in PALR group 65.9% vs. 50.9% in non‐PALR group, *p* = 0.178; 5‐year RFS in PALR group 33.2% vs. 29.0% in non‐PALR group, *p* = 0.092, data not shown). However, the differentiation of HCC recurrences due to multicentric carcinogenesis, intrahepatic recurrence, and local recurrences after LA is difficult in clinical practice. Nevertheless, PALR group consisted of recurrence cases and it was natural that the prognosis after hepatectomy was worse than non‐PALR group. Incidentally, in the present study, OS rate showed no significant difference between PALR and non‐PALR groups when it was evaluated at the time of initial treatment of HCC (10‐year OS 55.2% vs. 45.8%; *p* = 0.246). A future challenge will be to compare the prognosis of the patients who underwent hepatectomy or not for recurrence after LA.

Previously, ALBI grade, tumor number, tumor diameter, vascular invasion, and degree of liver fibrosis have been considered risk factors for poor prognosis after hepatectomy.^25^, ^26^, ^27^ he Cox‐multivariate analyses in the present study selected almost similar poor prognostic factors as previous study. In addition, serosal invasion was selected as an independent predictor of long‐term prognosis. However, since many reports that evaluated prognostic factors for HCC patients have not included serosal invasion in analyses,^25^, ^26^, ^27^ further study is needed to evaluate the clinical prognostic impact of serosal invasion in HCC.

In specific to the PALR group, the tumor size 3 cm or larger was an independent prognosticator in the present study. The data regarding ablation such as the number of LA treatments or intervals between LA and hepatectomy had no impact on prognosis. More detail data about LA treatment or standardized LA procedure is essential to assess its impact for long‐term survival. However, some of the data were lacking in the present study and further prospective research is warranted.

Pathological serosal invasion was significantly more common in the PALR group than in the non‐PALR group in the present study. Furthermore, presence of history of LA was selected as an independent predictor of serosal invasion. In addition, serosal invasion was an independent factor related to a dismal prognosis with respect to both OS and RFS in the present study. There have been a few previous reports concerning the effects of serosal invasion on prognosis in HCC. Sonohara et al. found serosal invasion to be a potent independent predictor of recurrence after hepatectomy.^28^ Previously, we showed that serosal invasion was an independent predictor of OS time on a par with the preoperative AFP level, hepatic cirrhosis, and the presence of invasion of the hepatic veins.^29^ We hypothesized that this poor prognosis might be affected by lymphatic‐mediated metastasis of HCC due to serosal invasion.^29^, ^30^ In addition, LA for a lesion close to the hepatic capsule might cause subcapsular lymphangiogenesis due to inflammation.^30^ Perihepatic lymphatic vessels not only link to cisterna chyli via hepatic nodes but also runs within portal tracts surrounded by Glissonean sheath.^30^ Therefore, lymphatic invasion might cause intrahepatic metastasis.^29^ Actually, although the present study did not show significant difference in the frequency of lymph nodes metastasis or peritoneal dissemination, aggressive intrahepatic metastasis which was mainly not indicated for local treatment (more than three lesions) was more frequent in positive serosal invasion group. Therefore, if there is a possibility of insufficient ablation for HCC, such as lesions close to the hepatic capsule, the use of this treatment method should be carefully weighed.

The limitations of this study are that it was a retrospective study, that it was conducted at a single institution, and that the sample size was limited. In the first place, the non‐PALR group consisted of HCCs with no previous treatment, and it was natural that the prognoses were better than that of the PALR group, which consisted of recurrent cases. Moreover, the patients in the PALR group and non‐PALR group have had much heterogeneity and biases. However, the biological characteristics of tumors that recurred after LA treatment were identified by the present study. Large, multicenter studies including precise immunohistopathological examinations and investigations of gene mutations are warranted to clarify the clinical impact of previous LA treatment on patients undergoing liver resection.

## 5 CONCLUSION

Hepatectomy for recurrence after LA for HCC had the risk of poor prognosis and serosal invasion. When hepatectomy is performed for recurrence after LA, thorough postoperative follow‐up is important.

## AUTHOR CONTRIBUTIONS

**Yusuke Nishi:** Formal analysis; investigation; methodology; resources; software; validation; writing – original draft. **Katsunori Sakamoto:** Conceptualization; data curation; formal analysis; investigation; methodology; project administration; resources; software; supervision; validation; visualization; writing – original draft; writing – review and editing. **Mio Uraoka:** Data curation; writing – review and editing. **Tomoyuki Nagaoka:** Data curation; formal analysis; supervision; writing – review and editing. **Masahiko Honjo:** Data curation; formal analysis; writing – review and editing. **Kei Tamura:** Data curation; formal analysis; supervision; writing – review and editing. **Naotake Funamizu:** Writing – review and editing. **Kohei Ogawa:** Data curation; supervision; writing – review and editing. **Yasutsugu Takada:** Supervision; writing – review and editing. **Yuzo Umeda:** Supervision; writing – review and editing.

## FUNDING INFORMATION

This study did not receive any financial support.

## CONFLICT OF INTEREST STATEMENT

The authors declare no conflict of interest.

## ETHICS STATEMENT

Approval of the research protocol by an Institutional Reviewer Board: This study was approved by the institutional review board at Ehime University Hospital (2211013) and was conducted in accordance with the ethical standards laid down in the 1995 version of the Declaration of Helsinki (as revised in Brazil 2013).

Informed Consent: Informed consent was by the opt‐out principle, with general information posted on the study website, along with the opportunity to refuse participation.

Registry and the Registration No. of the study/trial: N/A.

Animal Studies: N/A.
